# Expression and Role of *Vitellogenin* Genes in Ovarian Development of *Zeugodacus cucurbitae*

**DOI:** 10.3390/insects13050452

**Published:** 2022-05-11

**Authors:** Dong Chen, Hong-Liang Han, Wei-Jun Li, Jin-Jun Wang, Dong Wei

**Affiliations:** 1 Chongqing Key Laboratory of Entomology and Pest Control Engineering, College of Plant Protection, Southwest University, Chongqing 400715, China; cdyoucancd@email.swu.edu.cn (D.C.); hhl199773@email.swu.edu.cn (H.-L.H.); liweijun201608@163.com (W.-J.L.); wangjinjun@swu.edu.cn (J.-J.W.); 2 Academy of Agricultural Sciences, Southwest University, Chongqing 400715, China

**Keywords:** melon fly, vitellogenin, hormone regulation, nutrition stress, RNAi, ovarian development

## Abstract

**Simple Summary:**

*Vitellogenin* (*Vg*) is the precursor of the yolk protein gene, which is crucial for insect reproduction. In this study, we identified four *Vg* genes in *Zeugodacus cucurbitae* (Coquillett). Their molecular characteristics and expression patterns were analyzed. The four genes are mainly expressed in the fat body tissue of adult female melon flies, and their expression is regulated by the juvenile hormone and ecdysone. Nutritional stress significantly down-regulated their expression, indicating that nutrition-dependent vitellogenic development occurs during ovarian development. RNAi-mediated inhibition of the expression of the four genes resulted in significantly delayed ovarian development in *Z. cucurbitae*. The results indicate that the four genes play an important role in the development of ovaries in *Z. cucurbitae*.

**Abstract:**

*Vitellogenin* (*Vg*) genes encode the major egg yolk protein precursor in arthropods. In this study, four *Vgs* were identified in *Zeugodacus cucurbitae* (Coquillett). Sequence analysis showed that four *ZcVgs* had the conserved Vg domain. Phylogenetic analysis indicated that four *ZcVgs* were homologous to the Vgs of Tephritidae insects. The temporal and spatial expression patterns of *ZcVgs* were analyzed by quantitative real-time polymerase chain reaction (RT-qPCR), and the four *ZcVgs* showed high expression levels in female adults, especially in the fat body. The expression of *ZcVg1* and *ZcVg3* was down-regulated by a low dosage (0.5 μg) of 20-hydroxyecdysone (20E), and *ZcVg2*, *ZcVg3*, and *ZcVg4* were up-regulated by a high dosage (1.0 and 2.0 μg) of 20E. The expression of *ZcVg1* and *ZcVg2* was up-regulated by 5 μg of juvenile hormone (JH), while all of the *ZcVgs* were down-regulated by a low and high dosage of JH. Expression of *ZcVgs* was down-regulated after 24 h of starvation and recovered to normal after nutritional supplementation. After micro-injection of the gene-specific double-stranded RNA, the *ZcVgs*’ expression was significantly suppressed, and ovarian development was delayed in *Z. cucurbitae* females. The results indicate that RNA interference of reproduction-related genes is a potential pest control method that works by manipulating female fertility.

## 1. Introduction

*Vitellogenin* (*Vg*) genes encode the major egg yolk protein precursor in arthropods. In insects, Vg is mainly synthesized in the fat body in a tissue-, sexual-, and stage-specific manner [1]. It is secreted into the hemolymph, transported to the ovary, and absorbed by the receptor in oocytes [2]. The insect Vg protein was first discovered in the cecropia moth, *Hyalophora cecropia*, as a female-specific protein precursor for vitellin or yolk protein [1]. The Vg protein is a large glycolipophosphoprotein. The molecular weights vary from 150 to 200 kDa for large subunits and 40 to 65 kDa for small subunits belonging to the large lipid transfer protein (LLTP) superfamily [3]. Vg protein sequences are evolutionarily conserved across insect orders except for Diptera [2]. The identification of insect Vgs has revealed that they share similar structural motifs, including an N-terminal lipid-binding domain (LPD_N) and a von Willebrand factor type D similar domain (VWD) [2]. As in most insects, the consensus cleavage site for the primary Vg gene product is conserved near the N-terminus and is flanked by polyserine domains [4]. The most striking characteristic of insect Vgs is the existence of polyserine tracts, which serve as good phosphorylation sites [4]. However, the function of Vg phosphorylation remains elusive. Glycosylation of Vg has an essential role in folding and subunit assembly to achieve the mature protein in the hemolymph and ovary [5]. Vg has been studied extensively in a wide group of vertebrates and invertebrates, including insects. The number of *Vgs* varies greatly in different species [4]. For example, three *Vgs* have been found in *Drosophila melanogaster* [6], *Aedes aegypti* [7], and *Plautia stali* [8], and two *Vgs* have been found in *Riptortus clavatus* [4] and *Periplaneta americana* [9].

Generally, Vgs in insects are categorized into four types [1]. In the first, Vg is synthesized in the fat body in a sex-biased manner and absorbed by the developing oocytes in the ovary, as in the Vgs in *Perillus bioculatus* [10]. In the second, Vg is produced in both the fat body and ovarian follicle cells in females and is included in the development of oocytes, as in the Vg in *Musca domestica* [1]. In the third, Vg is synthesized in the ovarian follicle cells and included in the development of oocytes, as in the Vg in *Bombyx mori* [4]. Finally, in the fourth, Vg is produced in a non-sex-biased manner in the fat body, secreted into the hemolymph, and absorbed in the development of oocytes, as in a 30 kD protein in *B. mori* [11]. However, *Vg* expression is not female-specific and can be expressed in smaller amounts in the males of some species, such as *Apis mellifera* [1].

In insects, Vg synthesis is regulated by multiple signals. The regulation of *Vg* in insects is directed by hormones at the transcriptional level [2]. The mechanisms of hormone-based regulation of *Vg* gene transcription in insects are generally classified into three categories [1]. In the first, *Vg* gene transcription is only regulated by the juvenile hormone (JH), such as in *Heliothis virescens* [12] and *Maruca vitrata* [13]. In addition to JH, insects with the second mechanism also need the ecdysone hormone to co-regulate *Vg* gene transcription, such as in *M. domestica* [14]. Insects with the third type of mechanism require JH, ecdysone, and other hormones to co-regulate *Vg* gene transcription, such as in *A. aegypti* [15]. Nevertheless, JH is not responsible for Vg synthesis in social insects, and it has a primary role in regulating the age-based division of labor among workers, such as in *Pogonomyrmex californicus* [16]. In addition to these hormones, Vg synthesis is also affected by nutritional signals [2]. The starvation of female beetles resulted in a block in Vg synthesis but not in the progression of primary oocyte development to the resting stage in *Tribolium castaneum* [17]. Vg is essential for the maturation of adult eggs and the development of embryos in insects [2]. RNA interference (RNAi)-mediated suppression of the *Cadra cautella Vg* gene curtails oogenesis in the almond moth, *C. cautella* [18]. The silencing of the *Vg1* or *Vg2* genes inhibits oviposition in the Chagas disease vector *Triatoma infestans* [19]. The down-regulation of *ClVg* and *ClVg-like* expression in *Cimex lectularius* leads to the atrophy of ovarian tissue and reduced oviposition [20]. In Lepidoptera, the silencing of the *Vg* gene in *Corcyra cephalonica* caused severely abnormal ovaries [21]. In addition, the Vg in *A. mellifera* has multiple functions, including labor differentiation, reproductive competition, and climate adaptation [22].

The melon fly, *Zeugodacus cucurbitae* (Coquillett) [23], is one of the most troublesome agricultural pests [24]. It is widely distributed, including in Africa, temperate Asia, and many Pacific islands [25]. The genome of *Z. cucurbitae* has been sequenced and released, providing sequence information for gene annotation and functional research [26]. In this study, four *Vgs* were identified in *Z. cucurbitae* from the genome. The expression levels of *ZcVgs* in multiple development stages and tissues were investigated by quantitative real-time polymerase chain reaction (RT-qPCR). The effects of JH, 20-hydroxyecdysone (20E), and starvation on the expression of *ZcVgs* were also evaluated. The expected roles of these *ZcVgs* were also explored using RNAi, revealing their potential as pest control targets.

## 2. Materials and Methods

### 2.1. Insect Rearing

The insect stock was collected from Haikou, Hainan Province, China, in 2016. The adults were reared in an environmentally controlled insectary, with a temperature of 26–27 °C, a relative humidity (RH) of 65–75%, and a photoperiod of 14:10 h (light: dark). As prescribed in previously published work, *Z. cucurbitae* larvae were reared on an artificial diet consisting of bitter gourd, yeast powder, corn, wheat flour, and sucrose; adults were fed a mixture of sucrose, yeast powder, and honey [27].

### 2.2. RNA Isolation and cDNA Synthesis

Same-aged females were cultured under the same conditions as the stock flies. Five-day-old virgin female adults were collected. The total RNA was isolated using TRIzol reagent (Invitrogen, Carlsbad, CA, USA) according to the manufacturer’s instructions. The RNA sample’s concentration was determined by a NanoDrop One spectrophotometer (Thermo Scientific, Madison, WI, USA), that is, the purity of RNA samples (OD260/280 and OD260/230). The genomic DNA contamination of 1 μg of total RNA was removed using an RQ1 RNase-Free DNase kit (Promega, Madison, WI, USA). The first-strand cDNA was synthesized using the PrimeScript^®^RT reagent Kit (TaKaRa, Dalian, China) and stored at −20 °C until use.

### 2.3. Molecular Cloning and Sequence Analysis

The homologous Vgs from *Bactrocera dorsalis* (AF368053.1, AF368054.1, and ARV91014.1) were used as the query sequences to find the Vgs in *Z. cucurbitae* with a BLAST search against the genome in the National Center for Biotechnology Information (NCBI) database. Four fragment candidates were downloaded for the full-length confirmation by PCR. The accession number for the melon fly genome was ASM80634v1. The gene-specific primers were designed by Primer Premier 5.0 (PREMIER Biosoft International, Palo Alto, CA, USA) (Table 1). The PCR amplification was run on a Bio-Rad PCR machine (Bio-Rad, Singapore). PCR was conducted with 25 μL of a mixture consisting of 12.5 μL of 2× Taq PCR Master Mix (Biomed, Beijing, China), 9.5 μL of RNase-free water, 1.0 μL of the cDNA template (described above), and 1.0 μL each of forward and reverse primers (10 μM). PCR amplification was performed using the following procedure: initial denaturation at 95 °C for 3 min, followed by 35 cycles at 95 °C for 30 s, 55 °C for 30 s, 72 °C for 1 min, and a final extension at 72 °C for 10 min. The product was detected by 1% agarose gel electrophoresis. A QIAquick PCR Purification Kit (QIAGEN, Dusseldorf, Germany) was used to purify the positive products. The target product was connected to a pGEM-T by pGEM^®^-T Easy Vector (Promega, Madison, WI, USA) and transformed into *E. coli* Trans 5α (TSINGKE, Beijing, China). Finally, positive clones were obtained through blue-white spot screening and then sequenced.

The conserved domains of ZcVgs were analyzed by the SMART online website (http://smart.embl-heidelberg.de, accessed on 16 August 2021) [28]. For the alignment and phylogenetic tree construction, the homologs of Vg in other insects were derived from the NCBI database by BLASTp. Multiple sequence alignment was performed using Clustal Omega and visualized with Jalview 2.10 software [29]. The unrooted phylogenetic tree was constructed by the neighbor-joining method with 1000 bootstrap replicates using MEGA7 software [30].

### 2.4. Spatio-Temporal Expression Revealed by RT-qPCR

Virgin female adults were reared separately and collected to analyze the expression pattern of *ZcVgs* on each day from 0 to 9 d, with four biological replicates. Total RNA and cDNA preparation was performed as described above. *Ribosomal protein subunit 3* (*Rps3*) and *ribosomal protein L13* (*Rpl13*) were selected as the internal reference genes to explore the expression patterns of *ZcVgs* at different developmental stages [31]. The tissue samples from the midgut, fat body, Malpighian tubules, and ovary were dissected from 5 d old virgin female adults to determine the expression pattern of *ZcVgs*. Four biological replicates were prepared, and each sample was dissected from 20 individuals. *α-Tubulin* (*α-Tub*) and *β-tubulin 1* (*β-Tub1*) were selected as the internal reference genes to analyze the expression in various tissues of female adults [31]. The primers for the RT-qPCR were designed by Primer Premier 5.0 (Table 1), followed by the amplification efficiency determination. After the primers were synthesized, the standard curve was calculated to evaluate the amplification efficiency. Efficiencies in the range of 90–110% were acceptable. The qPCR reaction was run on a CFX384 Optics Module (Bio-Rad, Singapore) using a NovoStart SYBR qPCR SuperMix Plus Kit (Novoprotein, Shanghai, China). RT-qPCR was conducted with 10 μL of a mixture consisting of 5 μL of 2× qPCR mixture, 3.5 μL of RNase-free water, 0.5 μL of cDNA, and 0.5 μL each of forward and reverse primers (10 μM). The reaction program was 95 °C for 2 min, followed by 40 cycles at 95 °C for 30 s, 60 °C for 30 s, and a final at 60 °C for 1 min, 95 °C for 10 min. Melting curve analysis at 60–95 °C was performed to ensure the specificity of each primer. The gene expression was analyzed using qBase plus software [32].

### 2.5. Hormone-Induced Expression

First, 20E (TCI, Shanghai, China) was diluted in ethanol (Chuandong, Chongqing, China) to produce a stock solution with a concentration of 20 mg/mL. Then, the stock solution was diluted in ethanol to 0.5, 1.0, and 2.0 μg/μL as the work solutions. More than 300 female flies that had emerged on the same day were collected. After that, 1 μL of each solution was dripped onto the pronotum of each female adult on day 4, that is, 0.5, 1.0, and 2.0 μg/fly [33]. After treatment, all females were reared and collected for total RNA isolation after 12, 24, and 48 h. Females with an equal amount of ethanol dripped onto them served as the control group.

The JH analog Methoprene (Sigma, St. Louis, MO, USA) was dissolved in acetone (Chuandong, Chongqing, China) to produce a stock solution with a concentration of 20 mg/mL. The stock solution was then diluted in acetone to 2.5, 5.0, and 10.0 μg/μL. Then, 1 μL of each solution was dripped onto the pronotum of each female adult on day 4, that is, 2.5, 5.0, and 10.0 μg/fly. After treatment, all females were reared and collected for total RNA isolation after 12, 24 and 48 h. The females treated with an equal amount of acetone served as the control group. The *Rps3* and *actin 3* (*Act3*) were preliminarily evaluated as the internal reference genes to explore *ZcVgs’* expression induced by 20E; *Rps3* and *α-Tub* were preliminarily evaluated as the internal reference genes to explore the expression of *ZcVgs* induced by Methoprene. The expression of *ZcVgs* was detected by RT-qPCR.

### 2.6. Nutritional Stress

A total of 150 4 d old virgin females were reared and divided into three groups. Fifty females in group I were normally raised with sufficient water and nutrition. The females in group II were provided water only. After 12, 24 and 48 h, the females in groups I and II were collected for total RNA isolation. The females in group III were starved for 24 h and then provided with an adult diet for supplementary nutrition for another 24 h. The females in groups III were collected for total RNA isolation. *Rps3* and *RPl13* were selected as the internal reference genes to explore *ZcVgs’* expression patterns under nutritional stress. The expression of *ZcVgs* was detected by RT-qPCR.

### 2.7. RNA Interference and Functional Analysis

Gene-specific primers of four *ZcVgs* were designed for dsRNA synthesis using the Primer Premier 5.0. After amplifying the target fragments of 577, 533, 489, and 571 bp in ORFs, the dsRNAs were synthesized and purified using a Transcript Aid T7 High Yield Transcription Kit (Thermo Scientific, Vilnius, Lithuania) according to the manufacturer’s instructions. The concentration of dsRNA was tested with a NanoDrop One spectrophotometer. The integrity of the dsRNA was detected by 1% agarose gel electrophoresis. More than 500 female flies that had emerged on the same day were collected. Based on the spatio-temporal expression pattern of melon fly *ZcVgs*, the 3 d old *Z. cucurbitae* females were injected with 2 μg (50 nL) of dsRNA into the last ventral segment using a micromanipulator M3301R (World Precision Instruments, Sarasota, FL, USA). The females who delivered dsGFP fragment (378 bp) were set as the control group. Four biological replicates were conducted. Total RNA and cDNA preparation was performed as described above. *Rps3* and *α-Tub* were selected as reference genes to normalize the expression of *ZcVgs* on day 5 after RNAi [31]. Ovaries were dissected to determine the size on day 5. Thirty samples were dissected in each treatment, and the ovarian area (mm^2^) was measured using a Digital MicroscopeVHX-5000 (KEYENCE, Itasca, IL, USA).

### 2.8. Statistical Analysis

Gene expression was analyzed using the qBase plus software [32]. The significant differences among spatio-temporal expression and gene expression induced by the hormones were analyzed by the one-way analysis of variance with the least significant difference method (*p* < 0.05). The gene-silencing efficiency and phenotype (ovarian size) were analyzed by an independent sample *t*-test (*p* < 0.05). All of the analyses were performed by SPSS Statistics 22.0 (IBM, Chicago, IL, USA).

## 3. Results

### 3.1. Sequence Analysis and Phylogenetic Analysis

**Figure 1:**
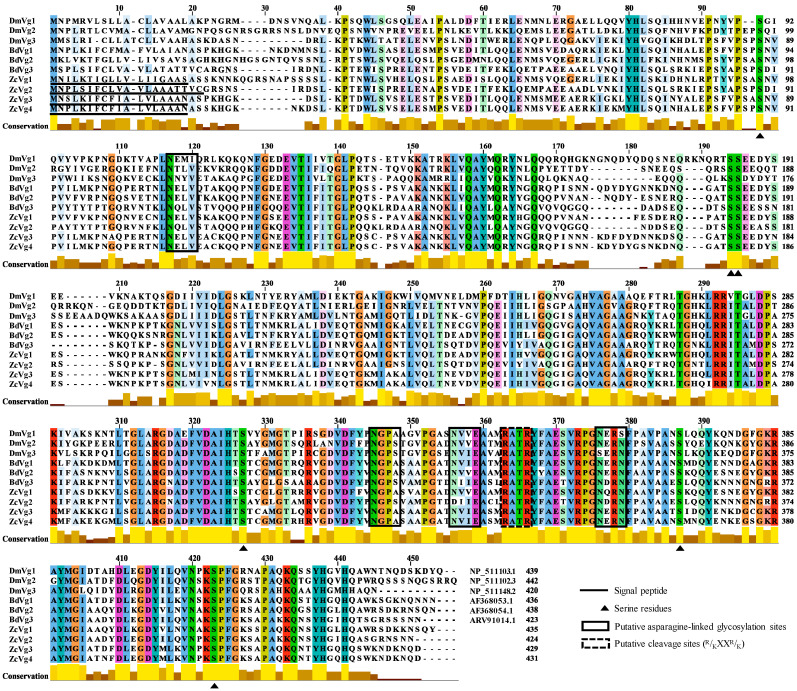
Sequence alignment comparison and analysis of ZcVgs and other insect Vgs. Multiple sequence alignment was generated using the amino acid sequences of Vgs from *Drosophila melanogaster* (Dm), *Bactrocera dorsalis* (Bd), and *Zeugodacus cucurbitae* (Zc). Putative cleavage sites (^R^/_K_XX^R^/_K_) are marked by a black dashed line box. Serine residues are marked by a black triangle under the alignment. Putative asparagine-linked glycosylation sites are marked by a black line box. The signal peptide is underlined by a black line. The height of the yellow/brown bars below the alignments indicates the degree of similarity of the amino acid above.

**Figure 2:**
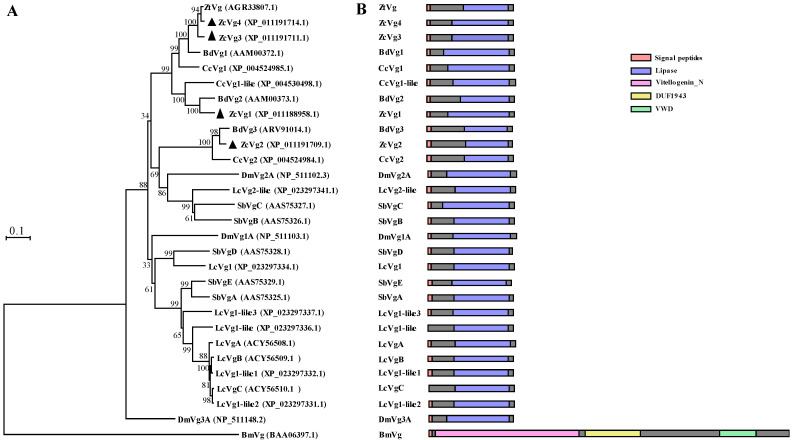
Phylogenetic analysis (**A**) and schematic comparison of protein structures (**B**) of Vgs in insects. The unrooted phylogenetic tree was generated with MEGA7 using the amino acid sequences of Vgs from *Z. cucurbitae* (Zc), *Drosophila melanogaster* (Dm), *Ceratitis capitata* (Cc), *Zeugodacus tau* (Zt), *Bactrocera dorsalis* (Bd), *Sarcophaga bullata* (Sb), *Lucilia cuprina* (Lc), and *Bombyx mori* (Bm). The black triangle indicates the ZcVgs. The accession number of each sequence is listed at the end. Signal peptide, lipase domain, vitellogenin_N domain, DUF1943 domain, and VWD domain are marked by pink, blue, purple, yellow, and green rectangles, respectively.

The full-length sequence confirmation showed that *ZcVg1, ZcVg2, ZcVg3*, and *ZcVg4* had open reading frames (ORFs) of 1308, 1275, 1290, and 1296 bp, respectively, encoding 435, 424, 429, and 427 amino acids, respectively. The sequence similarity of these four ZcVgs ranged from 53.90% to 92.81%. The sequence similarity of the four ZcVgs compared to other Dipteran Vgs ranged from 47.30% to 97.45%, while the sequence similarity of Vgs between Dipteran and *B. mori* Vgs ranged from 2.02% to 2.97% (Table S1). Sequences of the four genes (*ZcVg1, ZcVg2, ZcVg3*, and *ZcVg4*) were deposited in the NCBI GenBank database with the accession numbers OL546555, OL546556, OL546557, and OL546558, respectively. Sequence analysis revealed that all four *ZcVgs* possessed conserved domains with the LPD_N. They had putative cleavage sites (^R^/_K_XX^R^/_K_) and signal peptides. In addition, serine residues (the potential phosphorylation sites) and asparagines (NELV, NGPA, NVIE, and NERN–the glycosylation sites) were identified (Figure 1). The conserved lipase domains of ZcVg1, ZcVg2, ZcVg3, and ZcVg4 existed in 107–407, 91–399, 96–403, and 101–405 amino acids, respectively. The phylogenetic tree showed that ZcVg3 and ZcVg4 are most closely related to the Vgs of *Z. tau*, but ZcVg1 and ZcVg2 are most closely related to the Vgs of *B. dorsalis* (Figure 2).

### 3.2. Spatio-Temporal Expression of ZcVgs

**Figure 3:**
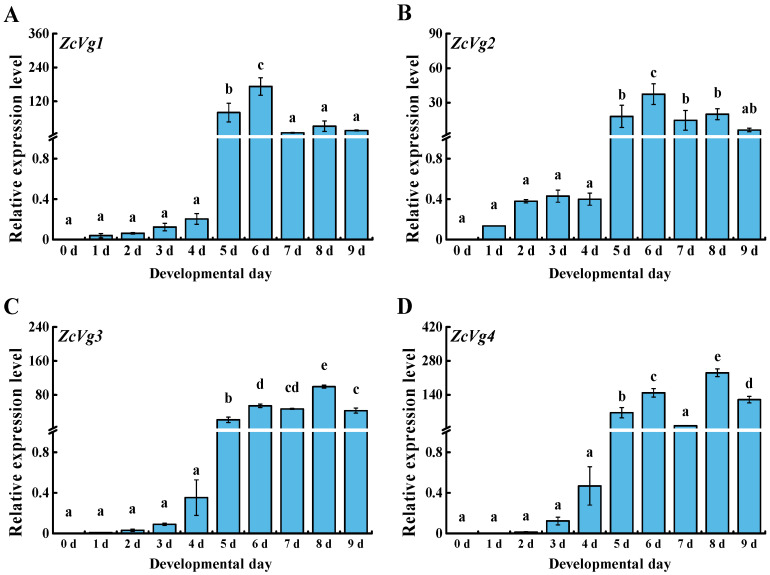
Expression patterns of *ZcVgs* in the development from eclosion to the sexual maturity of *Z. cucurbitae*. (**A**–**D**) Gene expression of *ZcVg1*, *ZcVg2*, *ZcVg3* and *ZcVg4*, respectively. The different letters on the bars represent the significant differences analyzed by SPSS 22.0 (*p* < 0.05, LSD in ANOVA).

The expression patterns of *ZcVgs* in different developmental stages showed that all of the genes were highly expressed from 5 to 9 d (Figure 3). In particular, *ZcVg1* and *ZcVg2* were significantly highly expressed in females from 5 to 9 d, with the highest expression at 6 d. *ZcVg3* and *ZcVg4* were significantly highly expressed in females from 5 to 9 d, with the highest expression at 8 d.

**Figure 4:**
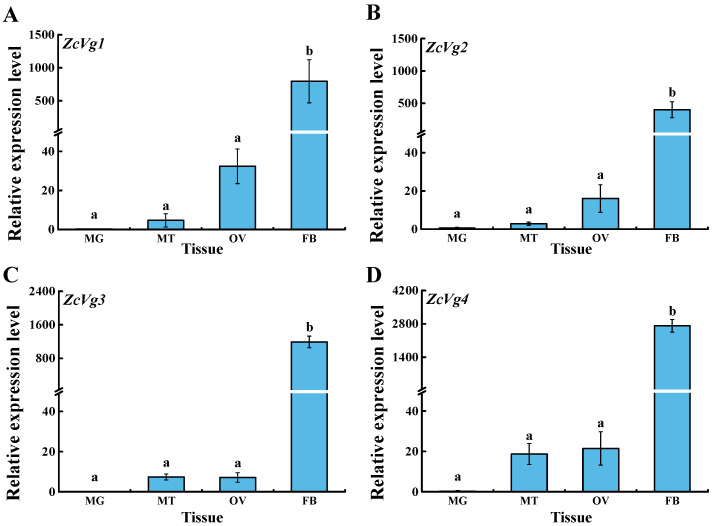
Expression patterns of *ZcVgs* in different tissues of 5 d old virgin female adults of *Z. cucurbitae*. (**A**–**D**) Gene expression of *ZcVg1*, *ZcVg2*, *ZcVg3* and *ZcVg4*, respectively. FB, MG, MT, and OV represent the fat body, midgut, Malpighian tubule, and ovary, respectively. The different letters on the bars represent significant differences (*p* < 0.05, LSD in ANOVA).

The expression patterns of *ZcVgs* in different tissues of female adults were analyzed. The results showed that the expression levels of *ZcVg1*, *ZcVg2*, *ZcVg3*, and *ZcVg4* in the fat body were significantly higher than those in other tissues (Figure 4). Therefore, the elevated transcript levels in the fat body are consistent with Vg biosynthesis in this tissue. In addition, these genes are also expressed in the ovary but with low expression levels.

### 3.3. Effects of Hormone Induction on ZcVgs’ Expression

**Figure 5:**
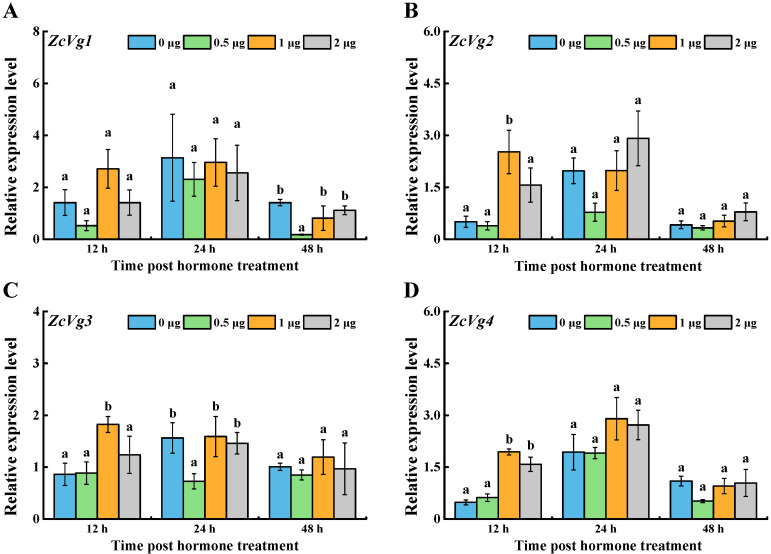
Effects of 20E on the expression of *ZcVgs* in *Z. cucurbitae*. (**A**–**D**) Gene expression of *ZcVg1*, *ZcVg2*, *ZcVg3* and *ZcVg4*, respectively. The different letters on the bars represent significant differences in the dose-dependent treatments at the same time-point (*p* < 0.05, LSD in ANOVA).

Four *ZcVgs* were induced by the exogenous hormone 20E with a different pattern (Figure 5). After 12 h, the expression of *ZcVg2*, *ZcVg3*, and *ZcVg4* in the 1 μg treatment group was up-regulated, and the expression of *ZcVg4* in the 2 μg treatment group was up-regulated. After 24 h, the expression of *ZcVg3* in the 0.5 μg treatment group was down-regulated. After 48 h, the expression of *ZcVg1* in the 0.5 μg treatment group was down-regulated.

**Figure 6:**
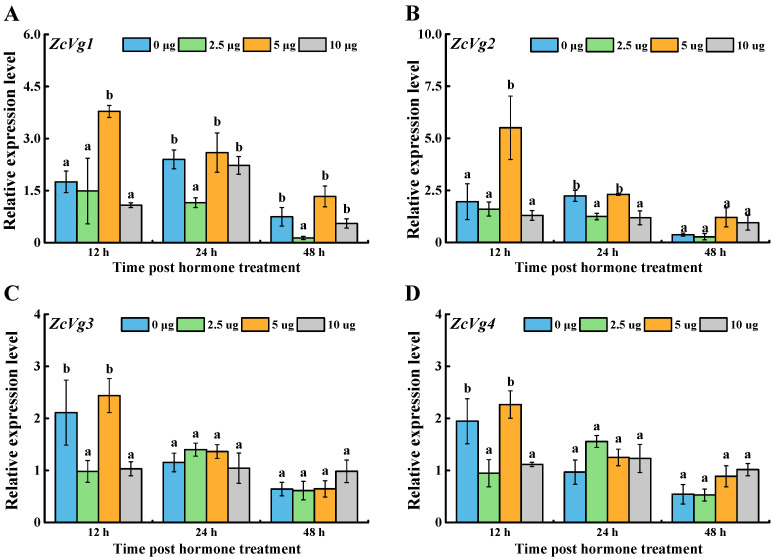
Effects of JH on the expression of *ZcVgs* in *Z. cucurbitae*. (**A**–**D**) Gene expression of *ZcVg1*, *ZcVg2*, *ZcVg3* and *ZcVg4*, respectively. The different letters on the bars represent significant differences in the dose-dependent treatments at the same time-point (*p* < 0.05, LSD in ANOVA).

Four *ZcVgs* were induced by the exogenous JH analogue Methoprene (Figure 6). After 12 h, the expression of *ZcVg1* and *ZcVg2* in the 5 μg treatment group was up-regulated, and the expression of *ZcVg3* and *ZcVg4* in the 2.5 μg and 10 μg treatment groups was down-regulated. After 24 h, the expression of *ZcVg1* and *ZcVg2* in the 2.5 μg treatment group was down-regulated, and the expression of *ZcVg2* in the 10 μg treatment group was down-regulated. After 48 h, the expression of *ZcVg1* in the 2.5 μg treatment group was down-regulated.

### 3.4. Effects of Starvation on ZcVgs’ Expression

**Figure 7:**
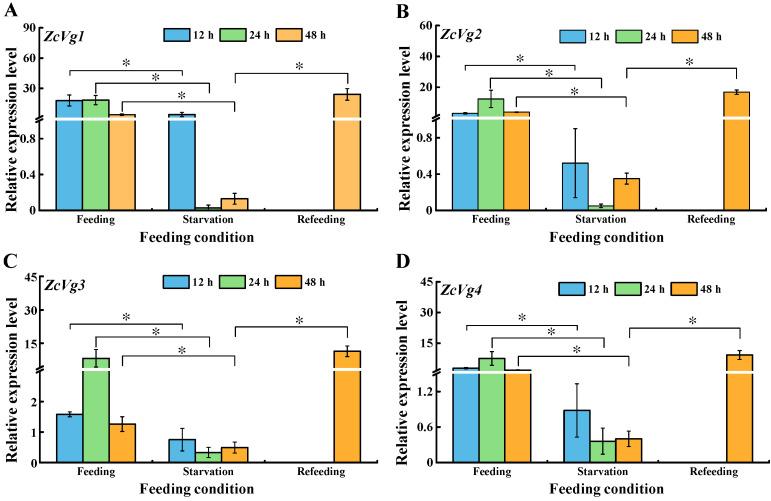
Effects of nutritional stress on the expression of *ZcVgs* in *Z. cucurbitae*. (**A**–**D**) Gene expression of *ZcVg1*, *ZcVg2*, *ZcVg3* and *ZcVg4*, respectively. The asterisks on the bars indicate significant differences analyzed by an independent sample *t*-test (* *p* < 0.05).

The results showed that the expression levels of four *ZcVgs* were down-regulated after 12, 24, and 48 h of starvation (Figure 7). After 12 h of starvation, the expression of *ZcVg1* was relatively less affected. Feeding after starvation can significantly induce *Vg* gene expression and return it to a normal level, or even a higher level than normal, such as in *ZcVg3*.

### 3.5. Effects of RNAi-Mediated Knockdown on Ovarian Development

**Figure 8:**
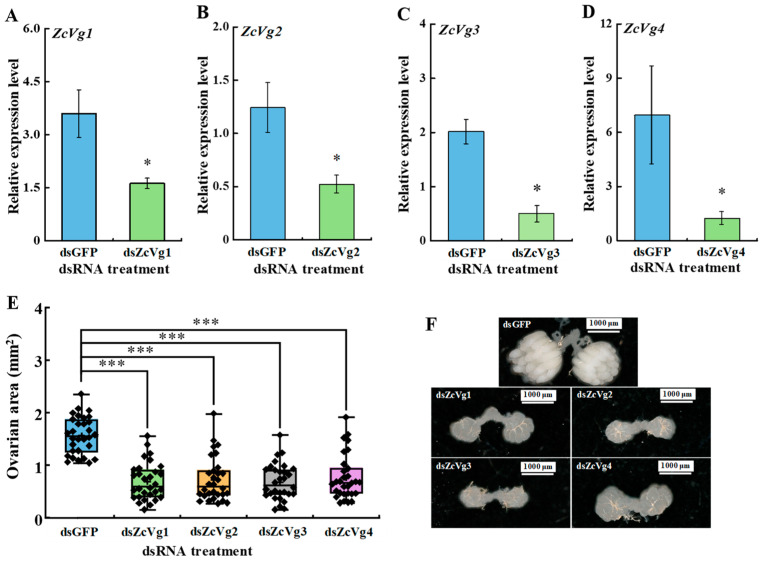
RNAi-mediated silencing affects the ovarian development of *Z. cucurbitae*. (**A**–**D**) RNAi efficiencies of four *ZcVgs*. (**E**) The ovarian size after RNAi treatments (*n* = 30). (**F**) Examples of the ovaries dissected in each treatment. A scale bar is provided in the upper right corner. The asterisks on the bars indicate significant differences between treatments and controls analyzed by an independent sample *t*-test (* *p* < 0.05, *** *p* < 0.001).

The results of RNAi showed that *ZcVg1*, *ZcVg2*, *ZcVg3*, and *ZcVg4* were significantly down-regulated by 54.9%, 58.0%, 75.1%, and 82.1%, respectively (Figure 8A–D). The ovarian area of the dsVgs treatment group was significantly smaller than that of the dsGFP control group, and ovarian development was significantly delayed compared to the control group (Figure 8E,F).

## 4. Discussion

The Vg hemolymph proteins are the precursors of yolk protein in the egg. The conserved Vgs have been studied extensively in various animals, both vertebrates and invertebrates, including insects [4]. In this study, we identified four *Vgs* in *Z. cucurbitae* based on the genome: *ZcVg1*, *ZcVg2*, *ZcVg3*, and *ZcVg4*. Multiple Vgs work together to ensure effective Vg production required for ovarian development. Variations in the *Vg* gene number in insects may reflect evolutionary selection and strategies to adapt to the environment [2]. Sequence analysis of *Vgs* revealed that the four *ZcVgs* possess conserved domains in Vgs and contain putative cleavage sites (^R^/_K_XX^R^/_K_), serine residues, and putative asparagine-linked glycosylation sites. These polyserine bundles are considered to be substrates for kinases. The negatively charged phosphoserine may affect protein solubility or chelate essential metal ions required for yolk production [34]. N-glycans may have a significant role in keeping this large, hydrophobic protein in the hemolymph to improve its transport to the ovary [5]. Vg, composed of the LPD_N for lipid binding, DUF1943, and vWFD, is highly conserved in most insect species except for aphids [35]. In addition, the length of the primary protein structure of the four ZcVgs is much smaller than that of most other order insects [36]. These much smaller Vgs are associated with the mammalian triglyceride lipase gene family [4]. Phylogenetic analysis indicated that ZcVgs are most closely related to the *Vgs* of other Tephritidae, such as *B. dorsalis* and *Z. tau*.

In this study, *ZcVg1*, *ZcVg2*, *ZcVg3*, and *ZcVg4* showed similar expression patterns, with the highest expression in the fat body. This expression pattern was similar to that of *B. dorsalis*, and Vg was synthesized in the fat body [37]. The VgR takes Vg up from the hemolymph and transports it into developing oocytes [4]. Therefore, based on the expression pattern of *Vgs* in *Z. cucurbitae*, the specific expression pattern of *Vg* indicates its function in synthesizing nutrition in the fat body. In contrast, low expression of *Vg* was observed in the male fat body and testis of *B. dorsalis* [37]. Analysis of the expression levels of *ZcVgs* in 0 to 9 d old female adults in *Z. cucurbitae* revealed that the mRNA level of *ZcVgs* was very high in 5 to 9 d old adults during the vitellogenic stage. Therefore, the expression of *ZcVg1*, *ZcVg2*, *ZcVg3*, and *ZcVg4* is correlated with the ovarian development of *Z. cucurbitae*. There are similar expression patterns in other insects, such as *Bemisia tabaci* [38] and *Actias selene* [39]. However, the up-regulation of *Vg* expression in some insects is observed earlier, with up-regulation beginning during the pupal stage. For instance, *Vg* expression is first detected during the late pupal stage in *Spodoptera litura* [40].

The expression of *Vgs* in insects is regulated by the ecdysone and JH, while the transcriptional mechanisms are different [1,41]. For instance, the transcription of the *Vg* gene in *Heliothis virescens* is only regulated by JH [1], while in *T. castaneum*, both JH and 20E are required for *Vg* gene expression [42]. After 20E induction, the expression of *ZcVg1* and *ZcVg3* was down-regulated by a low dosage, and the expression of *ZcVg2*, *ZcVg3*, and *ZcVg4* was up-regulated by a high dosage. Only the expression of *ZcVg1* and *ZcVg2* was up-regulated by 5 μg of JH, while all of the *ZcVgs* genes were down-regulated by a low and high dosage of JH. The following inferences are possible. The pathways activated by exogenous 20E after induction of a low dose and high dose were different in regulating *Vg* synthesis. A low dose and high dose of exogenous JH induced the same activation of the *Vg* synthesis pathway, but 5 μg of exogenous JH inducing the activation of the *Vg* synthesis pathway differs with a low dose or high dose. The pathway activated by low-dose exogenous 20E to regulate *Vg* synthesis has the same function as the pathway activated by low-dose and high-dose exogenous JH to promote *Vg* expression. The activation of the *Vg* synthesis pathway induced by exogenous 20E at a high dose was the same as that induced by exogenous JH at 5 μg, which inhibited *Vg* expression. The results of this study indicate that *ZcVgs* are regulated by the ecdysis hormone and JH, and the regulatory system is relatively complex. However, in the female American cockroach (*Periplaneta americana*), the *Vg* gene is activated by JH III and repressed by 20E via cis-regulatory elements in a dose-dependent manner [43]. JH III at doses of 0.1, 1, and 10 µg induced *Vg* synthesis in a dose-dependent manner, with doses as low as 0.1 µg being sufficient to significantly induce Vg synthesis in *Blattella germanica* [44]. The transcription of *Vg1* and *Vg2* in *P. americana* is induced by JH and inhibited by 20E in a dose-dependent manner [1], while both JH and 20E induce the *Vg* expression in *T. castaneum* [42]. Interestingly, in *Lymantria dispar*, JH induced or even down-regulated the expression of the *Vg* gene [1]. Both 20E and JH are extremely important for ovarian development by regulating the *Vg* expression. In *Nilaparvata lugens*, gene silencing using RNAi demonstrates the importance of JH signaling in ovarian development [45].

Nutrients play a pivotal role in insect vitellogenesis [2]. In this study, during ovarian development, the expression of *ZcVgs* was down-regulated after 24 h of starvation in melon flies, and the expression of *ZcVgs* could return to normal levels after nutritional supplementation. These results are similar to the effect of nutrition on the expression of *Vg* in *T. castaneum*, in which the starvation of female *T. castaneum* caused Vg synthesis to be blocked but did not cause oocyte development to enter the quiescent phase. Vg synthesis continued and oocyte development shifted from the quiescent phase to the mature phase after nutritional supplementation [17].

Gene silencing using RNAi is an effective method to explore gene function [46], and its use has proven successful in Diptera [47]. In this study, gene-specific dsRNA suppressed the *ZcVgs’* transcription and delayed ovarian development. These results indicate that *ZcVgs* play an important role in the development of the ovary in *Z. cucurbitae*. Interference with the expression of these four genes had a negative effect on ovarian development. This suggests that the expression of *ZcVg1*, *ZcVg2*, *ZcVg3*, and *ZcVg4* is required for ovarian development. It also demonstrates that multiple Vgs work together to ensure effective production of the Vg required for ovarian development. In addition, RNAi-mediated inhibition of *ZcVgs* showed an off-target effect as the conserved domain in sequences. It was speculated that the expression of one *ZcVg* was affected by the other *ZcVgs*. This off-target silencing of Vgs was also found in *Haemaphysalis longicornis*; after silencing each Vg, the expression of other Vgs was also significantly reduced, and the body weight and egg weight of ticks were significantly reduced [48]. In any case, the role of *ZcVgs* in ovarian development was validated by the RNAi work, showing potential targets for pest control. Since these four Vg sequences have a high similarity, this conserved sequence may be an excellent fragment target for pest control based on RNA interference. Previous studies have shown that the successful reproduction of insects depends on the production and deposition of Vg and the uptake of Vgs by VgR in developing oocytes [49]. The silencing of *Vgs* also leads to decreased reproduction in other insects and abnormal ovarian development. For example, suppressing *C. lectularius Vg* caused ovarian tissue atrophy and reduced egg production [20].

## 5. Conclusions

In this study, four *ZcVgs* were identified and cloned. Their molecular characteristics and expression patterns were analyzed. *ZcVgs* are mainly expressed in the fat body tissue of adult female melon flies. In addition, the expression of *ZcVgs* is regulated by JH and 20E in a dose-dependent manner. Nutritional stress significantly down-regulated the *ZcVgs’* expression, indicating that nutrition-dependent vitellogenic development occurs during ovarian development. RNAi-mediated inhibition of the *ZcVgs’* expression resulted in significantly delayed ovarian development. These results indicate that *ZcVgs* play an important role in the ovarian development of *Z. cucurbitae*. The results demonstrate that *Vgs* are potential candidates for a pest control method that works by suppressing their expression using RNAi to manipulate ovarian development.

## Figures and Tables

**Table 1:** Primers used in this study.

Gene	Upstream Primer (5′-3′)	Downstream Primer (5′-3′)	Purpose
*ZcVg1*	ATCTAGAGCACAATATGAATATACTGA	GGACACATTAGTATTGGCTGT	ORF cloning
*ZcVg2*	CCATGAATCCTTTAAGCATT	TAACCGTAGTTTAGTTGTTCGA	
*ZcVg3*	GATCATGAATTCACTGAAGATTT	TGTTAGCATATTAATCCTGGTTT	
*ZcVg4*	CATGAATCCGCTGAAGAT	TGTTAGCATATCAATCCTGGT	
*ZcVg1*	GCCAAATGATCGGCAAGACC	GAAGATCTTTGCGGGGTCCA	RT-qPCR
*ZcVg2*	TCTAGCCGTTCAAGCAGTCA	GTGTTGCCGGTTTGACG	
*ZcVg3*	GCTCCACACTCACCAACATG	CGAACATCTTGGCAGGGT	
*ZcVg4*	CACTCACCAACATGAAGCGT	TTAGCAGGGTCCAAAGC	
*Act3*	GAAACCTTCAACACACCCGC	CGGCCAAATCCAAACGAAGG	
*Rps3*	TAAGTTGACCGGAGGTTTGG	TGGATCACCAGAGTGGATCA	
*Rpl13*	GTTGTGCGTTGCGAGGAATT	GCTTGTCGTATGGTGGTGGA	
*α-Tub*	CGCATTCATGGTTGATAACG	GGGCACCAAGTTAGTCTGGA	
*β-Tub1*	GAATTGATGCGACTGGTGCC	CTGAATCCATGGTGCCAGGT	
*dsZcVg1*	taatacgactcactatagggTCATCTTTCCAAAATCGAT	taatacgactcactatagggACCAGTCCAGCGTTTGTA	dsRNA synthesis
*dsZcVg2*	taatacgactcactatagggCAGAAGCAACTCAATACGTG	taatacgactcactatagggCCAAATCAATGACCACTAAA	
*dsZcVg3*	taatacgactcactatagggACATGTCAATGGAAGAAGC	taatacgactcactatagggCAACATCGATAAGAGCTAAT	
*dsZcVg4*	taatacgactcactatagggATGTCAGTGGAAGAGGCC	taatacgactcactatagggCAACAATGTGGATGATCTC	
*dsGFP*	taatacgactcactatagggTGAGCAAGGGCGAGGAGCTG	taatacgactcactatagggTCGATGCGGTTCACCAG	

Note: T7 promoter sequence: taatacgactcactataggg.

## Data Availability

Data is contained within the article and Supplementary Material.

## Supplementary Materials

The following supporting information can be downloaded at: https://www.mdpi.com/article/10.3390/insects13050452/s1, Table S1. Identities of amino acid sequences of ZcVgs compared to other insect Vgs.
