# Supplementary material for: Expression and Role of Vitellogenin Genes in Ovarian Development of Zeugodacus cucurbitae
Source: Insects. 2022 May 11;13(5):452. doi: 10.3390/insects13050452 (PMC9143374; doi:10.3390/insects13050452)
Supplement: Supplementary file 1 [file insects-13-00452-s001.zip › insects-1666499-supplementary.pdf]

Supplementary materials:

Table S1. Identities of amino acid sequences of ZcVgs compared to other insect Vgs.

Identities (%) *	ZcVg 1	ZcVg 2	ZcVg 3	ZcVg 4	CcVg 1	CcVg1 -like	CcVg 2	ZtVg	SbVg E	SbulVg C	SbVg B	SbVg A	SbVg D	LcVg B	LcVg A	LcVg C	LcVg 1	LcVg 1-like	LcVg 1- like1	LcVg 2-like	LcVg 1- like2	LcVg1- like3	DmVg2 A	DmVg 1A	DmVg3 A	BdVg 1	BdVg 2	BdVg3	BmV g
ZcVg1	100	53.90	69.55	70.91	70.68	74.83	50.92	70.45	54.25	49.89	49.66	54.36	51.49	53.10	52.87	52.41	52.87	52.41	53.1	51.82	52.41	55.96	48.64	50.56	51.83	69.77	84.93	51.83	2.97
ZcVg2		100	55.56	56.02	55.94	51.14	85.21	56.25	53.65	49.42	52.18	52.10	53.38	53.29	52.82	53.05	53.72	53.76	53.29	50.23	52.11	51.15	50.79	49.77	48.48	54.00	52.16	91.53	2.47
ZcVg3			100	92.81	78.49	68.72	52.78	93.50	59.91	51.83	53.21	59.54	55.99	58.60	57.91	57.67	57.01	57.67	58.37	49.32	56.64	57.27	47.97	53.29	55.24	85.55	69.32	54.40	2.63
ZcVg4				100	82.65	70.32	53.24	97.45	58.24	52.28	52.98	59.31	55.53	58.00	57.08	57.08	57.01	56.84	57.77	52.26	55.68	58.18	47.30	52.61	54.29	88.30	69.09	54.63	2.81
CcVg1					100	71.53	55.25	81.51	57.67	50.91	53.42	58.50	57.67	59.04	57.67	56.98	58.58	54.00	58.81	53.83	54.92	57.82	49.77	56.11	52.63	82.42	69.09	54.11	2.69
CcVg1-like						100	52.51	68.95	50.80	48.30	49.54	52.28	52.40	53.55	51.95	51.72	54.34	51.72	53.55	49.66	51.72	52.26	48.53	56.11	52.40	69.41	73.47	51.14	2.97
CcVg2							100	53.70	51.64	49.54	51.95	51.52	51.86	51.88	51.17	51.64	51.74	52.11	51.64	52.16	50.00	50.69	50.34	47.06	47.44	53.32	51.03	83.33	2.36
ZtVg								100	58.47	52.74	53.21	59.31	56.22	58.24	57.31	57.31	56.55	57.08	59.16	52.49	55.92	58.41	47.75	52.61	54.52	87.84	68.64	55.32	2.81
SbVgE									100	50.35	50.23	83.37	62.06	76.18	75.94	75.47	59.67	75.94	76.65	50.00	74.23	71.72	47.96	55.33	53.65	56.88	52.74	53.52	2.58
SbulVgC										100	69.79	51.84	48.60	48.27	47.11	47.81	49.31	47.58	48.27	69.63	46.42	49.43	52.49	51.24	44.11	51.14	51.60	48.85	2.47
SbVgB											100	51.49	49.31	49.54	47.93	49.54	51.15	50.46	49.31	67.57	48.16	49.89	52.14	53.17	47.00	51.95	50.11	49.66	2.41
SbVgA												100	60.93	73.43	72.03	73.19	60.05	72.49	73.66	48.97	71.79	71.53	47.09	52.71	53.95	59.09	53.76	51.98	2.30
SbVgD													100	58.97	56.64	58.04	72.26	58.74	58.74	50.11	56.88	58.85	51.58	51.93	54.46	56.65	50.46	51.16	2.02
LcVgB														100	95.05	97.17	60.23	83.49	98.82	49.54	96.46	73.56	47.86	53.97	53.27	58.26	52.97	52.35	2.19
LcVgA															100	95.05	59.77	83.73	95.75	49.32	95.51	74.71	47.40	54.20	52.93	56.19	52.05	51.88	2.13
LcVgC																100	60.00	83.73	97.41	49.32	98.35	74.25	47.86	53.29	53.27	57.11	52.51	51.88	2.36
LcVg1																	100	60.00	60.00	49.09	60.23	61.84	54.30	56.33	53.70	59.95	52.05	51.97	2.58
LcVg1-like																		100	83.96	49.54	84.20	73.56	46.95	54.42	53.74	57.34	51.83	53.29	2.41
LcVg1-like1																			100	49.32	97.17	74.02	48.08	54.20	53.27	58.03	52.74	52.35	2.36
LcVg2-like																				100	49.32	50.57	51.91	50.33	45.33	51.35	50.45	50.80	2.69
LcVg1-like2																					100	74.02	47.86	53.97	54.10	56.19	52.28	51.41	2.30
LcVg1-like3																						100	49.10	54.71	53.08	59.41	55.13	50.69	2.41
DmVg2 A																							100	51.34	45.93	50.23	48.87	48.08	2.58
DmVg1 A																								100	51.47	55.33	51.91	48.19	2.97
DmVg3 A																									100	53.90	51.60	48.60	2.86
BdVg1																										100	68.41	53.09	2.86
BdVg2																											100	50.80	2.64
BdVg3																												100	2.24
BmVg																													100

* The identities were calculated with Align X program of Vector NTI.
